# Emerging Urinary Biomarkers and Innovative Technologies for the Early Detection and Personalized Management of Chronic Kidney Disease

**DOI:** 10.3390/ijms27083648

**Published:** 2026-04-19

**Authors:** Saltanat Moldakhmetova, Bikadisha Bimurat, Arailym Berdaly, Zhalaliddin Makhammajanov, Amankeldi A. Salybekov, Rostislav Bukasov, Abduzhappar Gaipov

**Affiliations:** 1 Department of Medicine, School of Medicine, Nazarbayev University, 010000 Astana, Kazakhstan; 2 Department of Chemical and Materials Engineering, School of Engineering and Digital Sciences, Nazarbayev University, 010000 Astana, Kazakhstan; 3 Regenerative Medicine Division, Cell and Gene Therapy Department, Qazaq Institute of Innovative Medicine, 010000 Astana, Kazakhstan; 4 Department of Chemistry, School of Sciences and Humanities, Nazarbayev University, 010000 Astana, Kazakhstan; 5 Division of Nephrology, Clinical-Academic Department of Internal Medicine, University Medical Center, 010000 Astana, Kazakhstan

**Keywords:** kidney, biomarkers discovery, chronic kidney disease, computational methods, data challenges, multi-omics integration, machine learning

## Abstract

Chronic kidney disease is a global public health concern, representing a critical global public health challenge with increasing morbidity and mortality rates. The disease is a long-term condition characterized by the progressive loss of renal function. Early detection of declining kidney health and timely intervention are crucial to slow disease progression and improve prognosis, mitigating complications, including cardiovascular events. Current diagnostic standards are unable to detect early stages of kidney disease, reflecting early signs of glomerular and tubular damage. This creates an urgent need to identify reliable biomarkers for early detection, prognosis and therapeutic monitoring of kidney diseases. Novel biomarkers, including urinary microRNA, exosomal components, proteomic signatures and integrated multi-omics profiles, facilitated by up-to-date technologies offer strong promise for enhancing early diagnosis, risk assessment and monitoring of the disease. We focus on the fundamental biological significance and clinical application of these markers, discussing a critical evaluation of novel methodologies and clinical evidence supporting their potential for earlier and more precise diagnosis. This review summarizes innovative urinary biomarkers and advanced analytical technologies that can provide a more comprehensive and accurate assessment of the kidney status towards early diagnosis, better prognosis and better quality of life for patients with chronic kidney disease.

## 1. Introduction

Chronic kidney disease (CKD) is a progressive long-term condition characterized by a gradual loss of kidney function that remains a serious public health challenge worldwide with an increasing morbidity and mortality rate [1]. The complex pathophysiological process of CKD involves glomerular, tubular, interstitial and vascular injury, ultimately resulting in fibrosis and renal failure [2]. Early diagnosis and active monitoring of patients with CKD enable timely intervention to significantly delay disease progression, reduce complications such as cardiovascular events, and improve survival rates.

CKD is a heterogeneous disease resulting in metabolic and hemodynamic complications. Despite nephroprotective treatments such as renin–angiotensin–aldosterone system inhibitors (RAASis) and new classes, including sodium–glucose cotransporter-2 inhibitors (SGLT2is), Mineralocorticoid Receptor Antagonists (MRAs), and Endothelin Receptor Antagonists (ERAs), the response to some drugs varies [3,4,5,6,7]. There is evidence that only 30–40% of patients respond to RAASis, leaving a large proportion of CKD patients with high risks of progression and complications [8]. Therefore, the development and validation of novel biomarkers, with further clinical application, are crucial initiatives to improve prognosis and predict treatment response [9].

**Figure 1:**
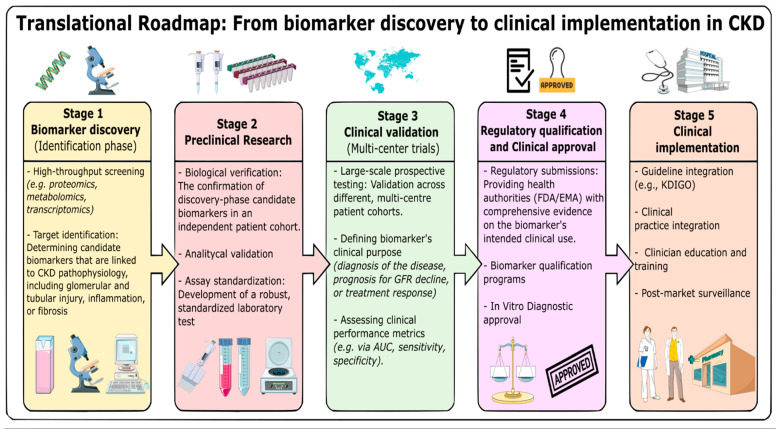
A translational roadmap summarizing the pathway from novel biomarker discovery to regulatory approval and clinical implementation. CKD—chronic kidney disease, GFR—glomerular filtration rate, AUC—Area Under the Curve, FDA/EMA—Food and Drug administration/European Medicines Agency.

Serum creatinine-based glomerular filtration rate (GFR) estimates and urine albumin remain the clinical standards for CKD detection and monitoring. However, the accuracy of GFR is limited due to physiological and external factors, including gender, age, diet, muscle mass and certain medications. Serum creatinine levels can also change independently, leading to inaccuracies in GFR estimation [10,11]. Despite a well-established graded association with CKD progression, albuminuria has limited sensitivity for early disease detection and primarily reflects glomerular injury, thereby incompletely capturing tubular and interstitial damage [12]. To address these diagnostic gaps, the integration of novel biomarkers and advanced technologies presents a promising approach. Figure 1 presents a translational roadmap outlining the critical steps for novel biomarker discovery to regulatory approval and clinical implementation (Figure 1). Biomarker’s strength to reflect dynamic processes, such as functional and pathophysiological changes, and to track responses to interventions refine the clinical picture missed by traditional tests [13,14,15]. In addition, the use of innovative multi-omics approaches, along with machine learning and artificial-intelligence-driven predictive models, enables a comprehensive analysis of complex biological and pathophysiological data, facilitating early detection and personalized patient care [16,17].

This review aims to comprehensively summarize the latest updates in emerging urinary biomarkers and innovative technologies for early detection of CKD, highlighting their biological significance, clinical application and possible integration into routine clinical practice and management of CKD. We conducted a literature search across the PubMed, Scopus, Web of Science and Google Scholar databases published in the last decade. Key search terms combined “CKD”, “urine”, “biomarkers”, “multi-omics”, “machine learning” and “artificial intelligence”. Studies in non-kidney disease focus, case reports and conference abstracts or studies lacking significant clinical biomarker data were excluded. Our primary focus is the assessment of novel investigative methodologies and clinical evidence of their efficacy.

## 2. Novel Urinary Biomarkers and Innovative Technologies

### 2.1. Urinary Non-Coding Small RNAs as Biomarkers

#### 2.1.1. MicroRNAs as Emerging Biomarkers in CKD

MicroRNAs (miRNAs) are small, approximately 20–25 nucleotides, non-coding RNAs that function as regulators of gene expression [18]. They form base pairs and bind to complementary sites within the 3′-untranslated regions of target mRNAs [19] and use multiple mechanisms to modulate the expression of target genes. During translation, they reduce protein expression by inhibiting the initiation and elongation steps [20]. Furthermore, they promote sequestering of target mRNAs into cytoplasmic processing bodies and facilitate their degradation [21]. They also mediate transcriptional gene silencing through binding to the promoter region [22]. In the kidney, miRNAs regulate processes critical to CKD progression, including inflammation, fibrosis, and endothelial integrity [23,24]. They are stable in biological fluids, both in protein-bound and inside extracellular cargoes. Due to the possibility of measuring them non-invasively in urine or plasma, miRNAs have been largely studied as candidate biomarkers for CKD diagnosis, staging, and predicted outcomes [18]. The preference of urine as a primary diagnostic substrate is supported by the systematic analysis of Bidin et al., which evaluated 63 studies highlighting urine samples as more advantageous for both the early detection and longitudinal monitoring of CKD compared to blood [25].

#### 2.1.2. Urinary microRNAs Associated with CKD Progression

As crucial regulators and biomarkers in CKD pathophysiology, the miRNA family has been studied to understand their role in fibrotic remodelling of renal tissue. The pathogenesis of renal fibrosis is closely associated with activation of TGF-β/SMAD signaling [26]. While miR-21 from tubular epithelial cells functions as a potent pro-fibrotic signal through the PTEN/Akt and TGF-β/SMAD pathways, miR-29 family members (e.g., miR-29s) are often downregulated and have potent antifibrotic properties [27,28]. However, their clinical utility varies by bio-specimen source. For instance, urinary exosomal miRNAs such as miR-192 and miR-29c demonstrated a close correlation with CKD progression in patients with diabetic kidney disease (DKD) [29]. Table 1 demonstrates different miRNA biomarkers by their sources and roles in pathological signature in CKD (Table 1). Although individual studies highlight a potential value of different miRNAs, evidence suggest that accuracy of a panel of miRNAs outperforms single miRNAs due to the lack of specificity to distinguish between primary renal injury and systematic metabolic noise [30,31].

#### 2.1.3. Diagnostic and Prognostic Performance

As of now, no miRNAs are yet approved for CKD in clinical practice. However, there are some miRNAs that demonstrate strong validation signals. Urinary miRNA panels, especially those associated with DKD (miR-192 with miR-29c or miR-21/miR-30 family members), show the most consistent pooled diagnostic performance and appear closest to clinical translation, provided that clinical validation is obtained and extensive prospective, well-designed cohort studies are conducted [44,45,46]. Among candidate microRNAs, miR-192, validated in preclinical studies, demonstrated strong diagnostic accuracy for DKD and could serve as a biomarker in the rule-in/rule-out panel for early DKD [47]. However, while some urinary miRNA panels have reached the early validation stage, they have not yet undergone the rigouros multicenter validation stage.

Importantly, miR-21 went beyond preclinical studies, and the first randomized controlled clinical trial tested the effectiveness of Lademirsen as an anti-miR-21 therapy to prevent kidney function loss. Nonetheless, no significant differences in eGFR fall were seen between Lademirsen-receiving group and the placebo group, questioning the viability of direct therapeutic targeting [48]. Although a previous study in an animal model demonstrated the effectiveness of slowing CKD progression [49], there is a divergence in disease modifiers across species. This also suggests that there is a need to differentiate between biomarkers of disease progression and the true therapeutic target. Despite data confirming the contribution of microRNAs during the transition toward ESRD, the scope of research remains narrow, especially in bridging findings to clinical practice. Future work should prioritize the discovery and validation of microRNAs in well-designed, multicenter prospective cohort studies.

#### 2.1.4. Limitations and Challenges of miRNA Use

The clinical application of miRNAs faces several technical and methodological bottlenecks. The absence of a universal standard protocol of sample handling, storage and processing significantly hinders further development and leads to inconsistent results across studies [50]. miRNAs exist at very low concentrations in biological fluids, which makes their measurement challenging. This also results in the utilization of high-throughput technologies and laboratory infrastructure, which increases the study’s cost, particularly for microarray analysis of miRNA expression [51]. Further hurdles include isolating miRNA from various cellular sources, which makes the extraction process difficult due to complexity and tissue heterogeneity [52]. In addition, the source of cellular contamination in biological fluids is cells, their pellet and their sediments. Lysed cells, including erythrocytes, leucocytes and platelets, release high levels of intracellular miRNAs, which can mask the true expression profile, rendering this data irrelevant to the disease state [53]. In addition, experimental design issues, including small sample sizes and the need to analyze samples at a large scale to identify miRNA expression considering age, gender, race, kidney disease stage and demographic factors, affect the robustness of miRNA biomarker studies [36]. The generalizability and reproducibility of these results are limited by the inability to recruit sufficient cohorts that represent the full spectrum of all these factors.

### 2.2. Urinary Extracellular Vesicle Biomarkers

#### 2.2.1. Definition and Importance of Exosomes in Kidney Physiology

Extracellular vesicles (EVs) are membrane-enclosed nanoparticles that mediate intercellular communication facilitating the transfer of bioactive cargo proteins, lipids, and nucleic acids between cells [54]. While medium and small EV buds are released from the plasma membrane, exosomes (30–150 nm) originate from the endosomal pathway through a highly regulated process that regulates cargo sorting and vesicle formation [55,56,57,58,59]. Exosomes are produced by different cell types and are found in multiple biological fluids, such as blood, urine, saliva, pleural and cerebrospinal fluids, breast milk, and semen [60,61].

From a clinical perspective, a large amount of genetic information and molecules in exosomes are derived from parental cells, which contributes to heterogeneity [62]. The membranous structure of exosomes, owing to their lipid bilayer, protects their cargo from enzymatic degradation across various body fluids and can be further detected, serving as a biomarker of disease [59].

**Figure 2:**
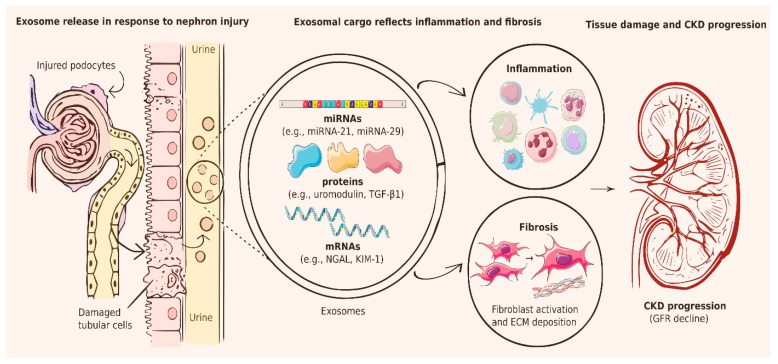
A schematic picture of exosome biogenesis and the clinical potential of exosomes as markers of CKD. Upon injury, podocytes and tubular epithelial cells release exosomes containing proteins, mRNA, or miRNAs. Exosomal cargo (e.g., transforming growth factor-β (TGF-β), Wilms’ tumor suppressor one (WT1), kidney injury molecule 1 (KIM-1), neutrophil gelatinase-associated lipocalin (NGAL), miRNA-21, miRNA-29, and others) reflects fibrosis, inflammation, and extracellular matrix (ECM) deposition, connecting non-invasive urinary biomarkers with chronic kidney disease (CKD). The figure was created using Inkscape v1.4. Images adapted from Servier Medical Art (https://smart.servier.com), licensed under CC BY 4.0.

Exosomes are released from every segment of the nephron and, owing to the downstream flow of filtrate from proximal to distal segments, retain molecular information reflective of the entire urinary tract (Figure 2) [63,64,65]. Crucially, both the quality and molecular composition of these vesicles can be altered by pathophysiological shifts in the kidney [66]. This leads to turning them from regulators of homeostasis into mediators of inflammation, tissue injury and fibrosis [64,67]. Consequently, analysis of exosomes isolated using non-invasive biofluids, such as plasma and urine, commonly referred to as liquid biopsy, offers a promising approach for the early detection and monitoring of kidney diseases (Table 2).

#### 2.2.2. Exosome Expression in Glomerular Cells

Glomerular endothelial cells, mesangial cells and podocytes utilize exosomal signaling to modulate kidney pathophysiological processes. In an in vitro model, Wu et al. showed that high glucose induced glomerular endothelial cells to release exosomes containing *TGF-β1* mRNA. These exosomes mediated the endothelial–mesenchymal transition in podocytes, thereby contributing to the development and progression of glomerulosclerosis [87,88]. Similarly, miR-145-5p has been shown to induce podocyte apoptosis and activate the RhoA/ROCK pathway [89]. While these findings represent potential biomarkers, they remain in the discovery phase as their predictive value has yet to be confirmed in human clinical cohorts [68,84,90].

Several exosomal signatures have been transitioned into early validation [73,91]. A panel consisting of miR-21, miR-29c, and miR-150 can serve as a prognostic indicator of disease progression and detect early renal fibrosis in lupus nephritis, reflecting histological changes [75]. Furthermore, urinary exosomal *ELF3* and *let-7c-5p* have emerged as specific indicators of podocyte injury among patients with type 2 diabetic nephropathy [74,76].

Studies involving a larger patient population have begun to strengthen the prognostic value of specific miRNA. Profiling a DKD cohort revealed that increased expression of urinary *miR-21-5p* and decreased expression of *miR-30b-5p* were significantly correlated with a decline in kidney function [77]. These candidates may provide significant prognostic value for longitudinal monitoring of patients at early risk, potentially providing a more sensitive marker compared to traditional filtration metrics for early stages of CKD.

#### 2.2.3. Exosome Expression in Tubular Cells

Proximal tubular epithelial cells (PTECs) utilize exosomes not only for transporting sodium–hydrogen exchanger 3 to the distal tubular epithelial cells but also as vehicles for communication in intercellular signaling along the nephron, including between proximal and distal tubular segments [92]. Under pathological conditions, such as hypoxia, inflammation, and oxidative stress, injured PTECs secrete exosomes that convey profibrotic signals to recipient cells, thereby promoting kidney fibrosis [91,93,94].

Exosomes originate from mesenchymal stem cells and carry growth factors, anti-inflammatory cytokines and protective miRNAs that promote tubular repair and attenuate kidney injury [95,96,97]. Ceruloplasmin, which may rise in urinary exosomes before the onset of proteinuria, is identified as a marker of kidney damage [98]. While this suggests a potential window for early intervention in CKD, these markers remain exploratory but can provide the rationale for future diagnostic panels.

Several studies have reached early clinical validation by correlating with biopsy findings. For instance, reduced urinary exosomal mRNA expression of CD2-associated protein directly reflected the degree of tubulointerstitial fibrosis [71]. In individuals with DKD, elevated exosomal mRNA expression of uromodulin expression by PTECs was significantly correlated with markers of poor outcomes, including albumin creatinine ratio and eGFR [72].

Urinary exosome mRNA and microRNA in kidney transplant recipients reported a close association between kidney fibrosis and allograft rejection, moving closer to clinical monitoring [86,99,100,101]. Recent multicenter data has advanced several tubular miRNAs, including miR-21, miR-29c, miR-150, miR-205, and miR-19, in urine samples as promising exosomal biomarkers that demonstrated accuracy in evaluating interstitial fibrosis and tubular atrophy in deceased donor kidneys [83].

#### 2.2.4. Limitations and Challenges of Exosome Use

The utilization of urinary exosomes can offer a non-invasive approach to detect and monitor kidney injury and serve as biomarkers in early diagnosis of CKD. Nevertheless, most studies are at the discovery or early validation phases [82,86], and large-scale clinical validation has not yet been applied in clinical practice. Several major challenges pose hurdles for clinical translation of urinary exosomes. The isolation and purification protocol has not been standardized. Researchers utilize diverse methods across studies, including differential ultracentrifugation, size-exclusion chromatography, immunoaffinity capture, polymer precipitation, and microfluidic-based isolation [102,103,104]. This methodological heterogeneity results in several gaps in their clinical application. Due to different isolation methods yielding vesicles of different purity and concentration, the results are frequently incompatible across studies. Another main hurdle is exosome isolation, which is often a time-consuming and expensive technique [105]. Additionally, urinary contaminants, most notably high-abundance uromodulin, can co-isolate with vesicles, thereby confounding downstream analyses [106]. The diverse cellular composition of the kidney and urinary tract also requires advanced and sensitive analysis, which complicates research and clinical use of exosomal biomarkers [107]. Furthermore, urine sample collection and storage may impact the outcomes, resulting in data variability and limiting reproducibility [108]. Before urinary exosomes can be applied, harmonized protocols, robust quality controls, and prospective multicenter longitudinal validation studies are needed.

### 2.3. Proteomic Signatures and Multi-Marker Panels

#### 2.3.1. Mass-Spectrometry-Based Proteomics as a Diagnostic Tool

CKD is a multi-factorial disease with various injury pathways. The complex pathophysiological process of kidney disease, which involves multiple molecular pathways, such as inflammation, immune response, fibrosis, and tubular injury, requires methodologies that can provide unbiased and comprehensive protein profiling [109]. State-of-the-art mass spectrometry (MS) technologies enable researchers to study proteins in the context of their roles in kidney pathophysiology, including protein expression, modifications, and interactions. These technologies also enable analysis of the molecular basis in clinical specimens, such as urine, plasma, kidney tissue and single cells, providing valuable information on disease mechanisms and potential biomarkers [110]. Currently, the use of a single marker does not provide specific information on particular aspects of kidney injury. Thus, a multi-marker panel combining proteins measured by multiplexed proteomics assays enhances sensitivity and specificity for CKD detection and reflects clinically relevant biomarkers, especially at early stages of the disease [111,112].

To obtain unbiased, qualitative and quantitative protein-based datasets with further high-accuracy analysis, mass spectrometry (MS) has been successfully utilized in studies of CKD. MS-based methodologies are fundamental to CKD proteomics, with a focus on the role of liquid chromatography with mass spectrometry (LC-MS/MS) and capillary electrophoresis–mass spectrometry (CE-MS) [113,114]. These techniques can simultaneously detect, identify, and quantify thousands of endogenous and exogenous proteins and peptides with high sensitivity and specificity [115]. Recently, MS has been employed to catalogue the normal human proteome, revealing that 71% of all human proteins (*n* = 14,264) are expressed in kidney [116,117] (https://www.proteinatlas.org).

Studies have shown that abnormal post-translational modifications (PTMs) of proteins can induce inflammation, oxidative stress and fibrosis in kidney tissue [118]. Although several diagnostic approaches, such as Western blotting and enzyme-linked immunosorbent assays, are available to detect molecular changes, a vast spectrum of post-translational modifications remain challenging to characterize comprehensively due to the limitations of existing antibodies [119]. Conversely, MS-based analysis has demonstrated the advancement of identification and measurement of post-translational modifications of proteins [120]. Modern MS workflows allow for simultaneous identification, site mapping, and relative quantification of multiple PTMs across complex proteomes [121]. Therefore, these capabilities make MS a powerful approach for discovering PTM-based biomarkers, elucidating mediators of disease and potentially identifying therapeutic targets with relevance for personalized strategies in CKD prevention and intervention [122,123].

#### 2.3.2. Urine Proteomics in CKD

Urine, as a non-invasive test, is the preferred fluid for CKD proteomics, yielding peptide/protein panels with strong prognostic value. A prospective multicenter study of 1775 patients with CKD was conducted, analyzing the urinary proteome to detect 273 potential biomarkers and develop a classifier for early detection and prediction of adverse kidney outcomes [124]. This was also validated in DKD cohorts [125]. A recent discovery study in advanced DKD populations (type 1 and type 2) identified complement pathway enrichment as the leading signal, showing strong associations across kidney outcomes, methods and diabetes subtypes with further confirmation by tissue proteomics, single-cell/spatial transcriptomics and validation in early-stage in three cohorts [126]. Urinary proteomics further enables early diagnostics in various renal diseases, including drug-induced nephropathy [127], IgA nephropathy [128], autosomal dominant polycystic kidney disease [129,130] and lupus nephritis [131,132,133].

The following Table 3 demonstrates the current landscape of glomerular and tubular biomarkers, highlighting their performance metrics and diagnostic accuracy (AUC).

As shown in Table 3, proteomic classifiers such as CKD273 currently represent the most advanced stage of transplational maturity. This result is likely due to the completion of large-scale multicenter longitudinal studies that provide the statistical significance required for clinical application.

#### 2.3.3. Limitations and Challenges of MS-Based Proteomics Use

Although current proteomics technologies represent powerful, high-throughput approaches for the discovery of CKD biomarkers, their application is limited by several technical, methodological, and clinical challenges. The wide dynamic range of protein abundance in urine and blood complicates MS analysis: low-abundance proteins are difficult to detect, whereas high-abundance proteins such as albumin and immunoglobulins can mask the presence of other clinically relevant proteins [134,135]. In addition, different proteomic platforms and analytical workflows often exhibit limited overlap in protein coverage, reducing reproducibility across studies. The prolonged time required for sample processing and data analysis in large-scale cohorts further constrains the clinical utility of MS-based proteomics. Moreover, candidate biomarkers require extensive validation in large, well-characterized patient populations over extended follow-up periods to achieve clinical relevance. Finally, the high cost of advanced instrumentation, particularly high-mass-accuracy MS platforms, remains a significant barrier to the widespread adoption of proteomics technologies in routine clinical practice [114].

### 2.4. Integration of Multi-Omics and Machine Learning

#### 2.4.1. Introduction to Multi-Omics

New advanced high-throughput technologies have been developed to capture a multifaceted understanding of the biological system [136]. Multi-omics approaches permit integrated analysis of multiple molecular layers, including genomics, transcriptomics, proteomics, and metabolomics, thereby linking genotype to phenotype and revealing the molecular networks that underlie complex diseases [137]. Several studies have investigated inherited kidney diseases using genomic testing [138,139,140,141,142], which may lead to practical applications of their findings, such as prevention and possibly interventional strategies [143]. Transcriptomic analysis in the context of kidney diseases can determine active molecular pathways using single-cell RNA sequencing in order to monitor tissue injury and repair [144,145,146,147,148]. Proteomics and metabolomics complement these approaches by profiling proteome and small-molecule signatures that reflect biochemical pathway activity. Large-scale metabolomics studies have identified candidate metabolites associated with early diabetic and chronic kidney disease progression [149,150,151]. Together, integrated multi-omics analyses improve mechanistic understanding, enhance prognostic and predictive accuracy, and shift the focus from treating late clinical manifestations toward identifying molecular drivers amenable to prevention and targeted intervention.

#### 2.4.2. Machine Learning Approaches for Multi-Omics Data

**Figure 3:**
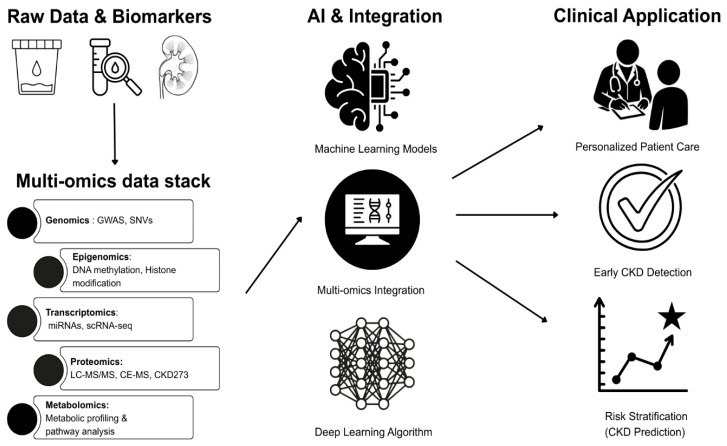
A schematic overview of the integration of multi-omics and machine learning. Usage of raw data in multi-omics: genomics (a genome-wide association study (SWAS), single-nucleotide variants (SNVs), epigenomics (DNA methylation and histone modification), transcriptomics (microRNAs (miRNA), single-cell RNA sequencing (scRNA-seq)), proteomics (liquid chromatography and mass spectrometry (LC-MS/MS), capillary electrophoresis–mass spectrometry (CE-MS), CKD273), and metabolomics (metabolic profiling and pathway analysis). Integration of artificial intelligence (AI) and machine learning models to improve early CKD detection and risk stratification that can be applied in clinical practice.

The machine learning (ML) approach is crucial for analyzing data from a large-scale set of samples, managing their complexity and heterogeneity. Different algorithms, such as supervised classifiers, unsupervised clustering and deep learning models, can help to detect molecular signatures with high precision (Table 4) [152,153,154,155]. Integration of ML in multi-omics enables the prediction of disease progression, anticipation of treatment response and identification of prognostic markers [154,156]. Selection of appropriate ML strategies is essential for successful multi-omics analysis, affecting the efficiency, robustness and clinical utility of the results (Figure 3) [157]. Depending on study objectives, various methods can be used in CKD research, including Multi-Omics Factor Analysis (MOFA), Data Integration Analysis for Biomarker discovery using Latent variables for Omic studies (DIABLO), random forest (RF) and deep learning (DL) [156,158].

Robust external validation remains essential yet challenging for ML models due to concerns about their interpretability and generalizability. Steyerberg et al. advocate using internal and internal–external validation over external testing, which provides only a “snapshot” in time, while internal validation can prevent overfitting by confirming pattern learning [159]. Conversely, independent database validation better detects site-specific biases missed by internal validation models [160]. However, external validation of ML showed insufficiency for ensuring patient safety [161,162,163]. Dataset shift from local differences (equipment, demographics, coding) often leads to success in a high-resource center but failure in a different clinical environment. Recurring local validation is currently considered a more reliable model, shifting responsibility for AI directly to the local healthcare system. This allows for overcoming many shortcomings of external validation by addressing the human–computer interface risk [164]. By using local explainability tools during regular checks, clinicians can verify that the model uses relevant local features [165,166].

The use of quantitative scoring of transcriptional characteristics has enabled exploring the mechanisms of progression deeply by uncovering specific gene expression motifs and molecular signatures in kidney diseases (Table 4) [167,168]. This can also serve as a marker of the therapeutic effects of drugs. Moreover, to evaluate the status of a transplant kidney, different ML approaches have been employed to enhance risk stratification and early identification of acute renal transplant rejection [169,170]. Additionally, studies have been conducted to develop new biomarkers of CKD through artificial intelligence algorithms, showing promising results in the assessment of CKD progression and complications among those patients [171,172,173,174,175]. However, a urine biomarker discovery study has been controversial when analyzing different tubular biomarkers in CKD [176]. This can be explained by heterogeneous etiologies of CKD and by different methodologies used in models. This highlights the main challenges and limitations in ML with multi-omics data.

Risk stratification and prognostic models can significantly improve timely intervention and the management of life-threatening conditions such as acute kidney injury (AKI). Applications of ML used in AKI predictive models assess the risk of the events, including in-hospital mortality, prolonged stay and unplanned readmission [177,178,179,180]. This tool is a potential approach to improve detection and reduce patient mortality rates among patients. The risk of ESKD in individuals with IgA nephropathy was assessed utilizing an artificial neural network and ML [181]. Furthermore, studies in DKD populations demonstrated the effectiveness of using ML and multi-omics data to classify and predict the prognosis [182,183,184]. While the findings identified high-risk patients for predicting their time-to-event endpoint which can help to initiate early prevention and intervention, it is important to note that most of these studies currently reside in the discovery or early validation phases.

**Table 4:** Summary of recent studies using different ML models to detect biomarkers of chronic kidney disease.

Studies, First Author, Year	ML Model Tested	Clinical Phenotype	Sample Type	Sample Size	Candidate ML Tools	Main Findings
Xiao et al. [185]	Linear models (Elastic Net, Lasso regression, Ridge regression, Logic regression), a support vector machine, random forest, XGBoost, neural network model, k-nearest neighbor algorithm.	CKD	Blood tests and demographic features	551	Elastic Net, Lasso regression, Ridge regression and Logic regression	Online predictive tools for CKD progression
Chan et al. [184]	ML-derived prognostic risk score (KidneyIntelX™) combining electronic health records and biomarkers.	DKD	Plasma	1146	KidneyIntelX	Prediction tools for the progression of DKD
Bai et al. [186]	ML models, including logistic regression, naïve Bayes and random forest.	CKD	Blood tests and baseline characteristics	748	Logistic regression, naïve Bayes and random forest	A potential use for patient screening, evaluation of the CKD prognosis
Islam et al. [187]	Multiple ML algorithms, including XGBoost.	CKD	The CKD dataset (24 features including blood and clinical parameters)	400	XgBoost classifier	Early detection/diagnosis of CKD using ML approaches to analyze big data parameters
Arif et al. [188]	The Boruta feature selection, the k-nearest neighbors algorithm with a hyperparameter optimization using grid-search cross-validation.	CKD	The CKD dataset (24 features including blood and clinical parameters)	400	Developing a robust ML model	Detection early stages of CKD
Dubey et al. [189]	ML algorithms, including gradient boost, decision tree, k-nearest neighbor, random forest, histogram boost and XGBoost.	CKD	Clinical parameters and blood tests	400	Gradient boost, XGBoost, histogram boosting and k-nearest neighbor	Potential for early diagnosis of renal disease and predicting prognosis
Cao et al. [190]	Weighted Gene Co-Expression Network Analysis, cytoscape software and machine learning, enrichment analysis, ROC curves.	CKD with Non-Alcoholic Fatty Liver Disease (NAFLD).	Gene expression data	407	Weighted Gene Co-Expression Network Analysis, cytoscape software and machine learning, enrichment analysis, ROC curves	Identification of diagnostic markers in CKD patients with NAFLD, namely, 4 NAFLD-related genes (DUSP1, NR4A1, FOSB, ZFP36)
Mustafizur Rahman et al. [191]	Random forest, voting, bagging, adaptive boosting, gradient boosting decision tree, extreme gradient boosting, light gradient boosting, stacking, MICE Imputation, Borderline-SMOTE for imbalance, recursive feature elimination and Boruta methods.	CKD	Clinical tests	Not reported	Light gradient boosting	Detection of CKD at early stages to prevent progression
Gu et al. [192]	LC-MS-based widely targeted metabolomics technology.	CKD	Plasma	62	Widely targeted metabolomics technology	The use of ML on metabolomics data to distinguish between different CKD stages to identify 7 metabolites as potential diagnostic biomarkers and therapeutic targets
Ghosh & Khandoker [193]	Logistic regression, random forest, decision tree, naïve Bayes, and extreme gradient boosting, SHapley Additive exPlanations and Local Interpretable Model-agnostic Explanations algorithms.	CKD	Demographic, biochemical, and clinical data	491 patients: 56 CKD, 435 non-CKD	Extreme gradient boosting with SHapley Additive exPlanations and Local Interpretable Model-agnostic Explanations algorithms	Assessment of patient’s kidney condition with further management and prevention of CKD progression
Metherall et al. [194]	Random forest and artificial neural networks.	CKD	Blood tests and demographic data	400	Random forest	Improvement in screening of the population, increasing CKD detection, on-time management
Sohal et al. [195]	Decision tree, random forest, k-nearest neighbor, logistic regression, XGBoost and AdaBoost.	CKD	Blood, urine tests	400	K-nearest neighbor	Biomarker identification for early CKD diagnosis
Zhang et al. [175]	Least absolute shrinkage and selection operator and SVM.	CKD	Gene expression datasets of 53 kidney biopsies	61	Least absolute shrinkage and selection operator and SVM	Explores the potential of amino acid metabolism-related genes as novel biomarkers for both the diagnosis and progression of CKD

#### 2.4.3. Limitations and Challenges of Multi-Omics and Machine Learning

Lack and diversity of datasets, low sample size, data quality and measurement errors constitute important issues at the first points of every study of ML in multi-omics [196,197]. Selecting standard algorithms for ML and AI models to interpret the results is a challenge for improving transparency and trust in data [156]. Furthermore, there is a need to validate the biomarker’s clinical performance and implications [198]. Ethical concerns and privacy preservation remain controversial issues in the processing of sensitive genetic and molecular data [199]. Although the high cost and resource-intensity of ML and multi-omics slow down the process of active adoption of these approaches in research [200,201]. Introducing predictive AI, including ML models, helps to monitor CKD progression, thereby reducing the cost of treatment such as dialysis and transplantation [202,203].

## 3. Future Perspectives of Emerging Biomarkers and Technologies in CKD

The implementation of advanced high-throughput technologies in kidney disease research has substantially transformed the landscape of CKD diagnostics. However, successful translation of novel biomarkers into clinical practice requires rigorous standardization of assay methodologies, biomarker thresholds, and sample collection and handling protocols. Harmonization of these parameters across laboratories is essential to ensure the reproducibility and comparability of results, which are critical for informed clinical decision-making. In addition, large-scale validation studies in ethnically diverse populations are necessary to establish the predictive accuracy, robustness, and generalizability of emerging biomarkers. The development of clinical guidelines incorporating biomarker testing will further facilitate their integration into nephrology practice, thereby advancing precision diagnostics and individualized therapeutic strategies.

MicroRNAs have emerged as promising biomarkers for the early detection of CKD due to their stability, accessibility, and disease-specific expression profiles. Identification of the main drivers among these markers for CKD progression will provide a possibility not only for timely detection and intervention but also for therapeutic purposes. Targeting specific mRNAs to inhibit renal inflammation and fibrosis, or using miRNA mimics to modulate pathophysiological processes, is a particular focus. These approaches have shown promising results by reducing the level of kidney damage, fibrosis, and albuminuria in preclinical studies [204,205,206]. Targeted delivery of miRNA to kidney tissue using nanoparticles, polymeric systems and nanocarriers can provide organ-specific therapeutic effects while eliminating side effects [207].

Urinary exosomes represent another emerging source of biomarkers for CKD. Further development and optimization of protocols for exosome isolation and purification are needed to improve quality control and reproducibility, enabling standardization for use in large-scale clinical studies. Increasing interest in exosomal cargo has contributed to an increasing number of research studies involving proteomic analysis. Proteomics studies enabled the detection of a specific protein and its alterations associated with CKD progression [208]. In addition, an exosome’s ability to carry and deliver specific miRNAs can also be used as a therapeutic source to restore and overexpress these miRNAs or to suppress certain miRNAs [58].

Despite these advances, economic considerations remain a major barrier to the widespread clinical adoption of biomarker-based approaches. Multi-omics technologies, AI-based analytical models, and the need for repeated biomarker measurements are associated with substantial costs. Nevertheless, the transition from reactive to proactive nephrology by utilizing biomarkers for early detection and intervention can delay the requirement for dialysis, which is one of the most expensive chronic treatments globally [209,210,211]. In addition, targeted validation reduces the cost of biomarker testing via RT-qPCR panels or antibody assays, maintaining robust prognostic capability [210]. Ongoing progress in high-throughput sequencing, single-cell and spatial omics, and nanotechnology-driven biosensors presents significant opportunities to identify biomarkers with enhanced sensitivity and specificity as well as driving down marginal costs. These technologies have the potential to improve disease classification, enhance prognostic and predictive accuracy, and enable monitoring of treatment responses in CKD [212]. Future efforts should prioritize the development of cost-effective assays and diagnostic platforms that balance analytical precision with economic feasibility.

## 4. Conclusions

Novel biomarkers and advanced technologies enable healthcare providers to better understand CKD pathophysiology and molecular heterogeneity. This represents a forward step for precision nephrology. Predicted on an individual’s unique genetic profile, personalized medicine could improve disease outcomes and reduce side effects. Promising biomarkers, including urinary microRNAs, exosomal components, proteomic signatures, and integrated multi-omics profiles, facilitated by machine learning, have shown substantial potential to improve early detection, risk stratification, and personalized therapeutic targeting beyond traditional biomarkers such as eGFR and albuminuria. However, significant barriers impede the clinical translation of these biomarkers. The primary scientific challenge is robust validation in large, diverse patient cohorts. This should be followed by implementation studies demonstrating both clinical effectiveness and cost–benefit. Finally, addressing challenges, including assay standardization, regulatory approval, ethical considerations, and healthcare accessibility, will be critical for their widespread adoption.

## Figures and Tables

**Table 1:** Summary of urinary miRNAs in CKD.

miRNA	Sample Type	Level in CKD	Role in CKD Pathogenesis	Key References
miR-21	Urine, urinary exosomes, tissue	increased	Podocyte injury, renal fibrosis, albuminuria	[28,32,33]
miR-29 family (29a/29b/29c)	Urinary exosomes, tissue	decreased	Renal fibrosis, extracellular matrix regulation	[34,35]
miR-30 family	Urinary exosomes	decreased	Podocyte injury, DKD	[36,37]
miR-192	Urinary exosomes	decreased	DKD progression	[35,38]
miR-155	Urine	increased	Renal fibrosis, endothelial dysfunction, inflammation	[39,40]
miR-146a	Urine	variable	Inflammation, proteinuria, renal fibrosis	[41]
miR-210	Urine	increased	Increased apoptosis	[42]
miR-126	Urine	increased	Albumuria, inflammation	[43]

**Table 2:** Urinary exosomes as candidate biomarkers of CKD.

Biomarker Candidate	Origin	Sample Size, n	Findings	Clinical Application	Reference
Wilms’ tumor-1	podocytes	25	Early detection of podocyte injury and CKD progression	Not reported	[68]
NGAL	tubular cells	15	Early biomarkers of delayed graft function after kidney transplantation	Not reported	[69]
Exosomal miRNA-29c	tubulointerstitium	32	Markers of renal function and severity of histological fibrosis	Not reported	[70]
Exosome mRNA of CD2-associated protein	podocytes	32	Evaluation of kidney function and renal fibrosis	Not reported	[71]
Uromodulin mRNA	epithelial cells	242	Correlation with GFR levels among patients with DKD and type 2 diabetes	Not reported	[72]
miR-200b	non-proximal renal tubule	50	Biomarkers of renal fibrosis	Not reported	[73]
let-7c-5p	renal cells	63	Indicators of renal function and DKD progression	Not reported	[74]
Exosomal miR-21, miR-150, miR-29c	tubulointerstitium	45	Detection of early renal fibrosis and assessment of the art of functional decline in lupus nephritis patients	Not reported	[75]
Elf3	glomeruli	50	Indicators of podocyte injury in DKD and minimal change nephrotic syndrome	Not reported	[76]
miR-21-5p miR-30b-5p	renal cells	31—discovery cohort 55—validation cohort	Altered expression of miRNA may detect early damage of the kidneys and monitor response to treatment in patients with DKD	Not reported	[77]
miR-188-5p, miR-150-3p, miR-760, miR-3677-3p, miR-548ah-3p, miR-548p, miR-320e, miR-23c miR-133a-3p miR-153-3p	podocytes, tubular cells	15	Identification of novel pathways of DKD and prediction of the target genes	Not reported	[78]
miR-451	renal cells	48	Early markers of renal injury and CKD	Not reported	[79]
Alpha1-Antitrypsin	renal tubular epithelial cells	147	Early markers of DKD prior to microalbuminuria	Not reported	[80]
N-osteopontin	tubular cells		Promotion of fibroblast proliferation and activation, acceleration of renal fibrosis	Not reported	[81]
hsa-miR-4488, hsa-miR-4532, hsa-miR-21-5p, hsa-miR-155-5p, hsa-miR-210-3p, hsa-miR-223-3p, hsa-miR-31-5p, hsa-miR-373-3p	renal epithelial cells	260	Early detection of acute rejection in kidney transplant recipients	Validation study	[82]
miR-21, miR-29c, miR-150, miR-205, miR-19	renal cells	109	Potential as biomarkers to evaluate the severity of interstitial fibrosis and tubular atrophy after kidney transplantation	Not reported	[83]
Podocalyxin and Nephrin	podocytes	Time points: 10 week (*n* = 18), 6 months (*n* = 25) 12 months (*n* = 14)	Possible long-standing and persistent renal damage	Not reported	[84]
Exosomal miR-136-5p	renal damaged cells	64	Upregulation of the marker of kidney damage and CKD progression among patients with DKD	Not reported	[85]
IL32, B2M, CXCL11, PGK1	renal cells	226	Identification and differentiation of patients with early signs of antibody-mediated rejection and T-cell-mediated rejection	Not reported	[86]

**Table 3:** Comparative diagnostic performance of urinary CKD biomarkers.

Biomarker Category	Representative Examples	Bio-Specimen	Performance (Sensitivity, Specificity, and AUC),	Reference
Exosomal miRNAs	let-7c-5p, miR29c-5p, miR-15b-5p	Urine	sensitivity, specificity—n/a, AUC: 0.818, 0.774, and 0.818	[74]
Exosomal miRNAs	miR-21, miR-29c	Urine	84.5%, 69.0%, 0.762, 98.3%, 67.9%, 0.795	[83]
Proteomic Classifiers	CKD273	Urine	85%, 100%, 0.96	[124]

## Data Availability

No new data were created or analyzed in this study. Data sharing is not applicable to this article.

## Abbreviations

The following abbreviations are used in this manuscript:

AKI	Acute kidney injury
Akt	Alpha serine/threonine-protein kinase
AUC	Pooled area under the curve
B2M	Beta-2-Microglobulin
CE-MS	Capillary electrophoresis–mass spectrometry
CKD	Chronic kidney disease
CXCL11	C-X-C Motif Chemokine Ligand 11
DIABLO	Data integration analysis for biomarker discovery using latent variables for omic studies
DL	Deep learning
DKD	Diabetic kidney disease
ERAs	Endothelin receptor antagonists
ESCRT	Endosomal sorting complex required for transport
ESKD	End-stage kidney disease
ECM	Extracellular matrix
*ELF3*	E74-like ETS transcription factor 3
EV	Extracellular vesicle
GFR	Glomerular filtration rate
IL-32	Interleukin-32
FDA/EMA	Food and Drug administration/European Medicines Agency.
KIM-1	Kidney injury molecule 1
LC-MS/MS	Liquid chromatography with mass spectrometry
ML	Machine learning
MS	Mass spectrometry
MRAs	Mineralocorticoid receptor antagonists
miRNAs	MicroRNAs
MOFA	Multi-omics factor analysis
NGAL	Neutrophil gelatinase-associated lipocalin
PGK1	Phosphoglycerate Kinase 1
PTEC	Proximal tubular epithelial cells
PTEN	Phosphatase and tensin homolog deleted on chromosome 10
PTM	Post-translational modifications
RAASis	Renin–angiotensin–aldosterone system inhibitors
RF	Random forest
RhoA	Ras homolog family member A
ROCK	Protein serine/threonine kinase
SMAD	Suppressor of mother against decapentaplegic
SGLT2is	Sodium–glucose cotransporter-2 inhibitors
TGF-β	Transforming growth factor-β
WT1	Wilms’ tumor suppressor one
